# Correlation of tear cortisol levels with morphological and biomechanical parameters of keratoconus

**DOI:** 10.3389/fmed.2025.1648334

**Published:** 2025-10-09

**Authors:** Xiaorui Zhao, Tong Sun, Yifei Yuan, Yu Zhang, Yueguo Chen, Jing Hong

**Affiliations:** ^1^Department of Ophthalmology, Peking University Third Hospital, Beijing, China; ^2^Beijing Key Laboratory of Restoration of Damaged Ocular Nerve, Peking University Third Hospital, Beijing, China; ^3^Peking University Institute of Laser Medicine, Beijing, China

**Keywords:** keratoconus, cortisol, pathogenesis, morphological parameters, biomechanical parameters

## Abstract

**Introduction:**

Keratoconus (KC) is a vision-threatening corneal disorder predominantly affecting young males, significantly impairing their quality of life. We aim to evaluate tear cortisol levels in KC and compare with controls, and to determine the correlation of tear cortisol levels with morphological and biomechanical parameters of KC.

**Methods:**

Age- and sex-matched 42 eyes of 42 patients were enrolled. The levels of tear cortisol were obtained by liquid chromatography–tandem mass spectrometry. Pentacam HR and Corvis ST II were used to detect the corneal morphological and biomechanical parameters. Spearman correlations between tear cortisol levels and corneal parameters were calculated for all patients.

**Results:**

Comparing with the control group, the level of tear cortisol significantly increased in the KC group (1660.95 [1175.01–2408.81] vs. 945.60 [550.36–1699.32], *p* = 0.023). It was positively correlated with Belin-Ambrosio Display D value (BAD-D), inferior–superior value (IS-value), keratoconus index (KI), Pentacam random forest index (PRFI), Corvis biomechanical index (CBI), and negatively correlated with central corneal thickness (CCT) and thinnest corneal thickness (TCT). No significant association was found between tear cortisol levels and maximum K value (Kmax), tomographic and biomechanical index (TBI), and stress–strain index (SSI).

**Conclusion:**

Our findings demonstrate that tear cortisol levels are significantly associated with some corneal morphological and biomechanical parameters in KC, suggesting its potential role as a pathogenic factor, thereby providing new insights into the exploration of disease pathogenesis.

## 1 Introduction

Keratoconus (KC) is a progressive bilateral corneal ectatic disorder characterized by corneal thinning, irregular astigmatism, and visual impairment (1). Corneal morphological and biomechanical assessments are critical for the early diagnosis and monitoring of disease progression in KC. Commonly evaluated morphological parameters include central corneal thickness (CCT), thinnest corneal thickness (TCT), maximum K value (Kmax), Belin-Ambrosio Display D value (BAD-D), inferior–superior value (IS-value), keratoconus index (KI), and Pentacam random forest index (PRFI) (2–5). In more advanced stages of KC, reduced corneal thickness, increased corneal curvature, and greater morphological deviation are typically observed. Among the biomechanical parameters, the most widely studied include Corneal Biomechanical Index (CBI), Tomographic Biomechanical Index (TBI), and Stress–Strain Index (SSI). In severe KC, CBI and TBI values are typically elevated, whereas SSI, which reflects corneal stiffness, tends to be decreased (6).

Numerous hypotheses have been proposed to explain the pathogenesis of KC, including genetic predisposition, oxidative stress, immunological disorders, corneal stroma proteolytic degradation, mechanical injury, and environmental pollution (7, 8). However, the precise pathophysiological mechanisms underlying KC remain incompletely characterized. Emerging evidence indicates that steroid hormones exert pleiotropic effects on corneal homeostasis. Melatonin demonstrates antioxidant efficacy and alleviates ocular surface inflammatory response in dry eye disease (9). Women undergoing *in vitro* fertilization treatment, with serum estradiol levels increased dramatically (10 to 50x), were found to have significant improvement in ocular symptoms and tear film associated with dry eye disease (10). Regarding cortisol, previous studies have investigated the association between hair cortisol concentration and KC, suggesting a positive correlation (11, 12). Dutta et al. (13) documented bilateral KC with right corneal scarring in a 52-year-old male diagnosed with adrenal myelolipoma. However, the relationship between tear cortisol levels and the morphological and biomechanical parameters of KC has not been systematically investigated. This study bridges this gap by establishing associations between tear cortisol levels and morphological and biomechanical characteristics in KC.

Cortisol, the primary glucocorticoid secreted by the adrenal cortex, regulates blood glucose levels, modulates the endocrine and immune systems, combats stress, and promotes the metabolism of fats, proteins, and carbohydrates (14, 15). Cortisol quantification is feasible in multiple biofluids, including serum, saliva, urine, and tears (14, 16). The substrate for cortisol synthesis is cholesterol, which is converted to pregnenolone (P5) and then undergoes a series of enzymatic reactions to generate progesterone (P4), 17α-hydroxypregnenolone (17-OHP5), 17α-hydroxyprogesterone (17-OHP), 11-deoxycortisol (11-DF), 21-deoxycortisol (21-DF), and ultimately cortisol (17–20). Liquid chromatography–tandem mass spectrometry (LC–MS/MS) methods, a specific and selective method, were used for the simultaneous quantification of the steroids.

We aimed to evaluate the tear cortisol levels in KC using LC-MS/MS, to investigate their associations with morphological and biomechanical parameters of KC.

## 2 Materials and methods

### 2.1 Ethical approval

This cross-sectional study was approved by the Ethics Committee of Peking University Third Hospital and followed the tenets of the Declaration of Helsinki. Informed written consents were obtained from all subjects.

### 2.2 Participants

The sample size calculation for this study was performed using PASS 15.0, with the primary outcome measure being the difference in tear cortisol levels between the KC group and the control group. Based on the mean concentration and standard deviation of tear cortisol levels, and assuming an alpha error of 0.05 and a power of 0.90, the calculated sample size required for statistical significance was 20 participants per group.

This study consecutively enrolled 42 participants from the Peking University Third Hospital Eye Center. Patients with bilateral KC were included in the KC group (*n* = 20), and those with bilateral mild-to-moderate myopia undergoing laser vision correction were included in the laser vision correction group (LVC) (*n* = 22). All patients were newly diagnosed and had not used any ocular or systemic medications within the 3 months prior to the visit. Both eyes underwent ocular examinations, with one eye randomly selected for enrollment. The diagnosis of KC was based on the stage I-IV on Amsler–Krumeich classification, with corneal tomography confirmation, and was confirmed by two experienced directors specializing in corneal surgery. Exclusion criteria included: ocular inflammation or infection, previous ocular surgery, ocular trauma, systemic hormone supplementation therapy, or connective systemic diseases. Contact lens discontinuation periods were required as follows: ≥ 14 days for soft lenses and ≥ 28 days for rigid gas permeable lenses.

### 2.3 Clinical examinations

All patients underwent a standardized bilateral ophthalmic evaluation, including medical history, corrected distance visual acuity, slit-lamp biomicroscopy, scheimpflug tomography (Pentacam HR; Oculus Instruments, Wetzlar, Germany), and dynamic biomechanical analysis (Corvis ST II; Oculus Instruments, Wetzlar, Germany). All examinations were conducted by the same certified technician under standardized conditions and using quality-controlled protocols to minimized bias. Corneal morphological parameters, including CCT, TCT, Kmax, BAD-D, IS-value, KI, PRFI, as well as biomechanical characteristics, including CBI, TBI, and SSI, were collected and analyzed.

### 2.4 Laboratory investigations

In this study, tear cortisol, P5, P4, 17-OHP5, 17-OHP, 11-DF, and 21-DF were quantified, with cortisol designated as the primary outcome measure. Considering the secretion rhythm of endogenous cortisol, tear sampling was consistently performed at the same time (9–10 a.m.). For the selected eye per participant, approximately 20 μL of tears fluid were collected from the inferior lateral meniscus using a sterile glass micropipette under slit-lamp guidance. The procedure was completed within a continuous 5-min period to minimize reflex secretion and ensure sample integrity for all participants. The samples were then stored at −80 °C until analysis. For LC–MS/MS analysis, 20 μL of each sample was aliquoted into a 1.5 mL Eppendorf tube, followed by the sequential addition of 20 μL of internal standard, 80 μL of ultrapure water, and 400 μL of tert-butyl methyl ether. The mixture was vortex-mixed for 5 min, then centrifuged at 12000 rpm for 8 min, and 350 μL of the supernatant was transferred to a 96-well plate. The extraction was repeated with tert-butyl methyl ether, the supernatants were combined, dried under nitrogen at room temperature, and dissolve in 40 μL of 10% acetonitrile solution for analysis. Tear steroid quantification was performed using the Waters ACQUITY UPLC I-Class/Xevo TQ-XS Absolute system (Waters Corporation, Milford, Massachusetts, United States). Tear extracts were analyzed on a Waters TQ-XS micro tandem mass spectrometer equipped with a Waters ACQUITY I-Class UPLC system. Analytical separation was performed using a Waters BEH C18 column (1.7 μm, 2.1 × 100 mm). The chromatographic flow rate was 0.3 mL/min. Nitrogen was used as the desolvation gas at 550 °C with a flow of 1,100 L/h. The lower limits of quantification for the analytes in tears were as follows: cortisol, 30 pg./mL; P5, 5 pg./mL; P4, 5 pg./mL; 17-OHP5, 10 pg./mL; 17-OHP, 2 pg./mL; 11-DF, 2 pg./mL; and 21-DF, 2 pg./mL. The recovery rates for all analytes ranged from 85 to 115%. The intra- and inter-assay coefficients of variation for all analytes were less than 15%.

### 2.5 Statistical analysis

All statistical analyses were conducted in SPSS (Version 26.0, IBM Corporation, Armonk, New York, United States) and GraphPad Prism (Version 10.0, GraphPad, San Diego, California, United States) with two-tailed tests and *α* = 0.05. The Shapiro–Wilk test was used to test for normality. Normally distributed data were expressed as mean ± SD and compared using the independent samples t-test between the KC group and the LVC group; non-normally distributed data were reported as median [IQR] and analyzed with the Mann–Whitney U test. Notably, as cortisol was the primary outcome, Bonferroni correction was applied when comparing the six other cortisol-related measures. The χ^2^-test was used in the analysis of the gender ratio. Spearman rank correlation analysis was used to explore the correlations between tear cortisol levels and different corneal parameters. *p* < 0.05 was considered statistically significant.

## 3 Results

Table 1 presented comparative analyses of demographic characteristics, tomographic indices, and biomechanical parameters between KC (*n* = 20) and LVC (*n* = 22) groups. The cohorts were age- and sex-matched (KC vs. LVC: 26.00 [23.00–29.50] vs. 23.50 [20.00–25.00] years; 63.64% vs. 60.00% male; *p* > 0.05) with comparable baseline characteristics. KC patients exhibited characteristic ectatic changes: reduced corneal thickness (CCT, TCT), elevated biomechanical indices (CBI, TBI, SSI), and abnormal tomographic markers (Kmax, BAD-D, IS-value, KI, PRFI).

**Table 1:** Patients demographics and clinical parameters.

	KC	LVC	*p* value
Gender (female/male)	8/12	8/14	0.808
Age (years)	26.00 [23.00–29.50]	23.50 [20.00–25.00]	0.073
CCT (μm)	481.50 [441.00–499.90]	547.00 [522.00–564.00]	<0.001
TCT (μm)	464.50 [401.50–491.50]	540.00 [517.00–563.00]	<0.001
Kmax (D)	54.75 [48.15–67.55]	44.50 [43.20–45.40]	<0.001
BAD-D	8.24 [3.64–16.03]	1.01 [0.55–1.83]	<0.001
IS-value	4.50 ± 3.24	0.10 ± 0.68	<0.001
KI	1.22 ± 0.16	1.02 ± 0.02	<0.001
PRFI	1.00 [0.90–1.00]	0.07 [0.02–0.30]	<0.001
CBI	0.99 [0.39–1.00]	0.00 [0.00–0.00]	<0.001
TBI	1.00 [1.00–1.00]	0.16 [0.03–0.50]	<0.001
SSI	0.67 ± 0.13	0.79 ± 0.12	0.003

The gender distribution was presented as male/female (χ2-test). Continuous data were expressed as mean ± SD for normally distributed data (t-test) or median [IQR] for non-normally distributed data (Mann–Whitney U test). LVC = laser vision correction; KC = keratoconus; CCT = central corneal thickness; TCT = thinnest corneal thickness; Kmax = maximum K value; BAD-D = Belin-Ambrosio Display D value; IS-value = inferior–superior value; KI = keratoconus index; PRFI = Pentacam random forest index; CBI = Corvis biomechanical index; TBI = tomographic and biomechanical index; SSI = stress–strain index.

Table 2 delineates tear steroids profiles quantified via LC–MS/MS, encompassing P5, P4, 17-OHP5, 17-OHP, 11-DF, 21-DF and cortisol. Tear cortisol level in KC group was higher than that in LVC group (1660.95 [1175.01–2408.81] vs. 945.60 [550.36–1699.32], *p* = 0.023). The tear levels of P5, P4, 17-OHP5, 17-OHP, 11-DF, 21-DF showed no significant between these two groups.

**Table 2:** Levels of cortisol-related hormones.

	KC	LVC	*P* value
Cortisol (pg/mL)	1660.95 [1175.01–2408.81]	945.60 [550.36–1699.32]	0.023
P5 (pg/mL)	168.97 [5.00–227.31]	161.46 [5.00–215.02]	0.856
P4 (pg/mL)	5.00 [5.00–5.00]	5.00 [5.00–5.00]	0.587
17-OHP5 (pg/mL)	173.35 [96.14–328.98]	126.91 [76.94–187.46]	0.247
17-OHP (pg/mL)	52.40 [32.35–75.40]	51.37 [38.69–96.49]	0.546
11-DF (pg/mL)	13.39 [7.03–34.52]	10.03 [5.50–16.33]	0.137
21-DF (pg/mL)	23.57 [2.00–28.96]	22.34 [2.00–33.46]	0.979

Continuous data were expressed as median [IQR] for non-normally distributed data (Mann–Whitney U test). The primary outcome was tear cortisol; comparisons of the six other cortisol-related hormones used the Mann–Whitney U test with Bonferroni correction. LVC = laser vision correction; KC = keratoconus; P5 = pregnenolone; P4 = progesterone; 17-OHP5 = 17α-Hydroxypregnenolone; 17-OHP = 17α-Hydroxyprogesterone; 11-DF = 11-Deoxycortisol; 21-DF = 21-Deoxycortisol.

**Figure 1:**
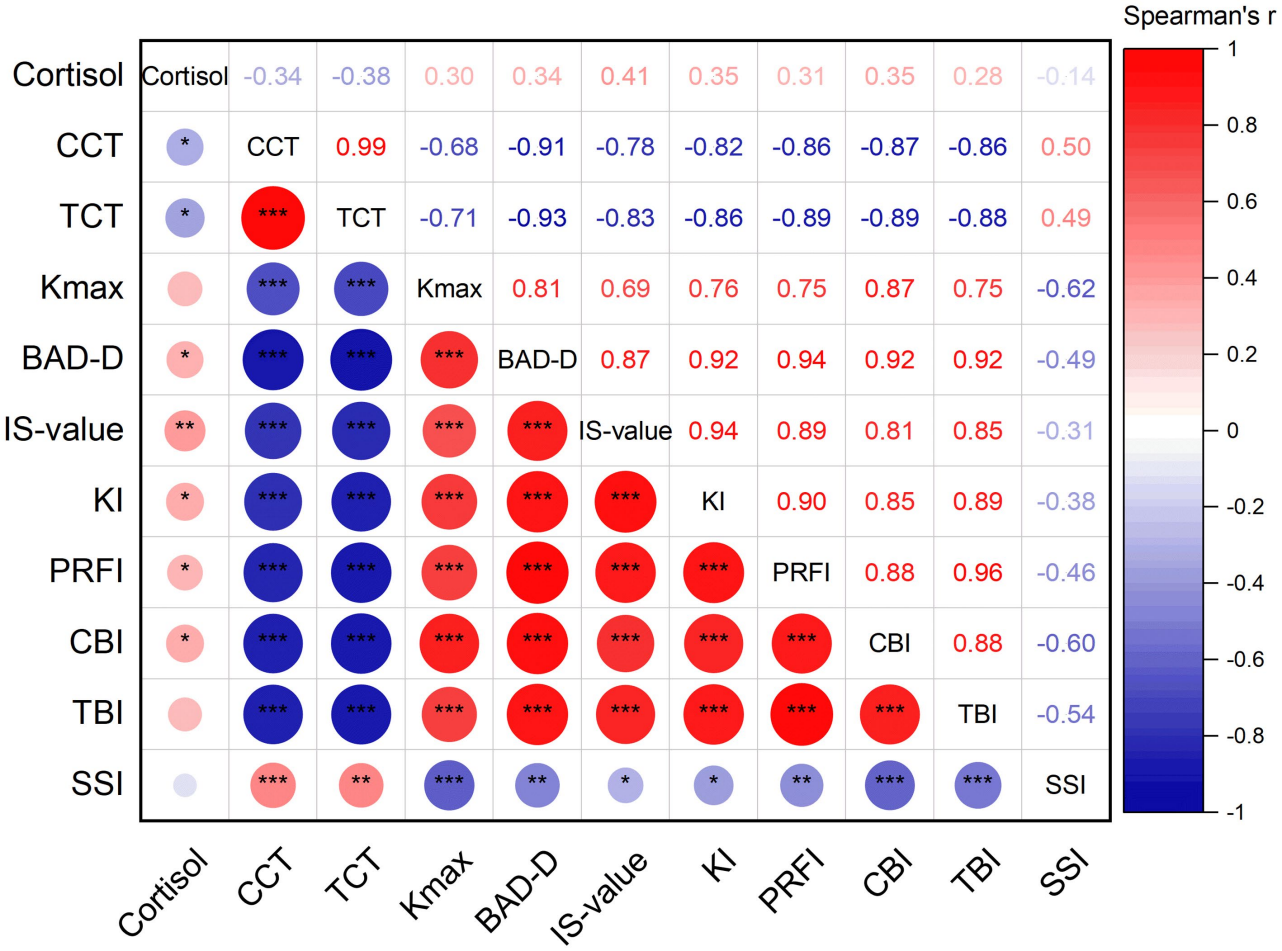
Spearman correlation analysis between cortisol, the corneal morphological parameters, and corneal biomechanical parameters for all patients (CCT: central corneal thickness; TCT: thinnest corneal thickness; Kmax: maximum K value; BAD-D: Belin-Ambrosio Display D value; IS-value: inferior–superior value; KI: keratoconus index; PRFI: Pentacam random forest index; CBI: Corvis biomechanical index; TBI: tomographic and biomechanical index; SSI: stress–strain index; *, *p* < 0.05; **, *p* < 0.01; ***, *p* < 0.001).

Spearman correlation analysis was used to find the relationships between the tear cortisol, corneal morphological parameters, and corneal biomechanical parameters for all patients (Figure 1). Among them, tear cortisol levels demonstrated statistically significant positive correlations with BAD-D (r = 0.34, *p* = 0.030), IS-value (r = 0.41, *p* = 0.007), KI (r = 0.35, *p* = 0.022), PRFI (r = 0.31, *p* = 0.047), and CBI (r = 0.35, *p* = 0.023). Conversely, significant inverse correlations were observed between tear cortisol levels and CCT (r = −0.34, *p* = 0.030) and TCT (r = −0.38, *p* = 0.014). No significant association was found between tear cortisol levels and Kmax (r = 0.30, *p* = 0.057), TBI (r = 0.28, *p* = 0.070), and SSI (r = −0.14, *p* = 0.391).

## 4 Discussion

This study revealed significantly elevated tear cortisol levels in patients with KC compared to those with mild-to-moderate myopia (control group). Cortisol levels were significantly positively correlated with parameters indicative of KC severity (Kmax, BAD-D, IS-value, KI, PRFI, CBI and TBI) and negatively correlated with parameters reflecting corneal structural integrity (CCT, TCT and SSI).

Cortisol’s role in ocular pathophysiology has been well-documented across multiple diseases, spanning anterior to posterior segment of ocular diseases. In Cushing’s syndrome, patients had significantly lower central macular thickness compared to those with normal cortisol levels, and increased cortisol levels were correlated with lesser central macular thickness and thicker central choroidal thickness (21). Endogenous hypercortisolism in Cushing’s syndrome confers a higher risk of ocular hypertension, which suggests cortisol screening should be considered in the assessment of glaucoma (22). Chronically elevated hair cortisol correlates with KC progression and epithelial thickness vatiation (23). Topical corticosteroids inhibit corneal wound strength in rabbits and humans (24). Larissa et al. (12) established progression KC as a hypercortisolemic state mediated through tear interleukin (IL)-6 level.

Our preliminary findings suggest an association between cortisol and KC progression, although the causal relationship remains unclear. We propose two hypothetical pathways.

First, cortisol may play a pathogenic role in KC development through the following two potential mechanisms: (a) Cortisol may exacerbate ocular surface oxidative stress and inflammation, ultimately worsening KC. Patients with Cushing’s syndrome exhibit profound oxidative imbalance, as demonstrated by elevated levels of plasma 15-F_2t_-Isoprostane compared to healthy controls (25). Concurrently, vitamin E levels were found to be reduced and were inversely correlated with urinary free cortisol levels (25). Exogenous cortisol provokes profound oxidative stress in human platelets, characterized by increased reactive oxygen species (ROS), elevated superoxide anions, and lipid peroxidation (26). This oxidative burst coincided with depletion of reduced glutathione, thereby compromising cellular redox homeostasis (26). Oxidative stress plays a critical role in the pathogenesis and progression of KC. KC corneas demonstrate sustained oxidative overload, with ROS and reactive nitrogen species levels significantly higher than those of healthy controls (27). Oxidative insult induces mitochondrial DNA (mtDNA) deletions and a reduction in mtDNA copy number in KC keratocytes (28). Cumulative oxidative damage triggers keratocyte apoptosis and disorganization of the extracellular matrix (ECM) (29). Mechanistically, oxidative stress may remodel the ECM by upregulating matrix metalloproteinase-2 and downregulating tissue inhibitor of metalloproteinases-1, thereby disrupting the architecture of corneal stromal collagen, particularly type IV and V collagen, and ultimately promoting the development of KC (30). Cortisol may modulate corneal stromal collagen homeostasis in KC through proinflammatory cytokines and matrix metalloproteinases (MMPs) expression. In coronary artery disease, evening cortisol levels have been shown to have strong positive correlations with the levels of total and active MMP-9, which are significantly elevated in the corneal epithelium and tears of KC. Serum inflammatory markers IL-6 was strongly positively correlated with the levels of evening cortisol, which also has been found elevated in KC (31). A previous study confirmed that in patients with progressive KC, levels of IL-6 in tear and cortisol in hair were significantly higher (12). However, this regulatory effect appears to be dose-dependent. A study found that low-concentration hydrocortisone increased the release of IL-6 and IL-8 in human corneal epithelial cell lines, while high-concentration hydrocortisone can inhibit their expression (32). (b) Cortisol might cause systemic metabolic disturbances leading to obesity, which alters corneal surface pressure gradients, thereby worsening KC. Elevated endogenous cortisol serves as a significant etiological factor not only for neuropsychiatric disorders but also for metabolic derangements, including metabolic syndrome (characterized by insulin resistance and dyslipidemia) and central obesity. Crucially, these pathological alterations may reciprocally exacerbate cortisol dysregulation, thereby establishing a self-reinforcing vicious cycle. Obesity has been implicated in modifying periocular anatomical architecture, including palpebral fat pad hypertrophy, orbital septal adipose expansion, and reduced orbital volume. These structural alterations amplify damage on the cornea through anterior corneal surface pressure gradient distortion induced by orbital space compression, intraocular pressure fluctuations secondary to retrobulbar adipose accumulation, and also chronic low-grade ocular inflammation (33, 34).

Second, patients with KC may experience significant psychological stress, which in turn alters the release of cortisol. Vision loss in patients with KC is a significant contributing factor to increased psychological burden. A psychiatric comorbidity burden was observed in KC cohorts: among 57 KC patients, 63.2% had anxiety disorders, 56.1% had depression, 10.5% had schizophrenia, and 1.8% had bipolar disorder, suggesting that there is an association between psychiatric disorders and KC (35). Cortisol, as one of the body’s primary stress hormones, is released in response to psychological stress. When an individual experiences psychological stress, the hypothalamus triggers the release of corticotropin-releasing hormone, which stimulates the anterior pituitary gland to secrete adrenocorticotropic hormone, ultimately leading to cortisol production by the adrenal cortex (36, 37). In patients experiencing their first episode of psychiatric disorders, heightened cortisol levels correlate with the severity of clinical symptoms, which suggests that psychiatric disorders and psychological stress are associated with cortisol levels (36, 38–40).

It is worth noting that patients with depression may exhibit behaviors or habits such as eye-rubbing or neglecting ocular care, which could exacerbate KC or accelerate its progression (33, 41–44). The mechanical effects of eye rubbing and their potential causative mechanisms may involve: elevated corneal temperature, thinning of the epithelial layer, heightened inflammatory mediator levels in pre-corneal tear fluid, dysregulated enzymatic activity, transient intraocular pressure surges, elevated tissue hydrostatic pressure, thixotropic reduction in ECM viscosity, transient displacement of matrix components from the corneal apex, fibril deformation and buckling induced by corneal indentation waves, biomechanical redistribution of curvature toward the cone apex, inter-fibrillar slippage at the conical apex, keratocyte alterations caused by mechanical stress and/or elevated hydrostatic pressure, alongside scar tissue development (45–51).

This study represents the first to quantify tear cortisol in KC using validated UPLC-MS/MS, revealing its great significance for exploring the role of cortisol in the pathogenesis of KC. This study has several limitations. First, the sample size of this study is relatively small. Second, the use of a mild-to-moderate myopic population as controls limits generalizability; future studies should consider enrolling patients without ocular or systemic comorbidities. Third, the study design is cross-sectional and correlational, which cannot establish causality or directionality. Further studies are warranted to elucidate the interplay between tear and blood cortisol levels at different time points, inflammatory mediators (e.g., cytokines), and oxidative stress markers in KC pathogenesis.

In conclusion, UPLC-MS/MS-based tear cortisol profiling identifies of KC progression, offering new insights into the exploration of the pathogenesis of KC.

## Data Availability

The raw data supporting the conclusions of this article will be made available by the authors, without undue reservation.
